# Supplementary material for: Deep splicing plasticity of the human adenovirus type 5 transcriptome drives virus evolution
Source: Commun Biol. 2020 Mar 13;3:124. doi: 10.1038/s42003-020-0849-9 (PMC7070027; doi:10.1038/s42003-020-0849-9)
Supplement: Supplementary file 1 — Supplementary Information [file 42003_2020_849_MOESM1_ESM.pdf]

Supplementary Figures

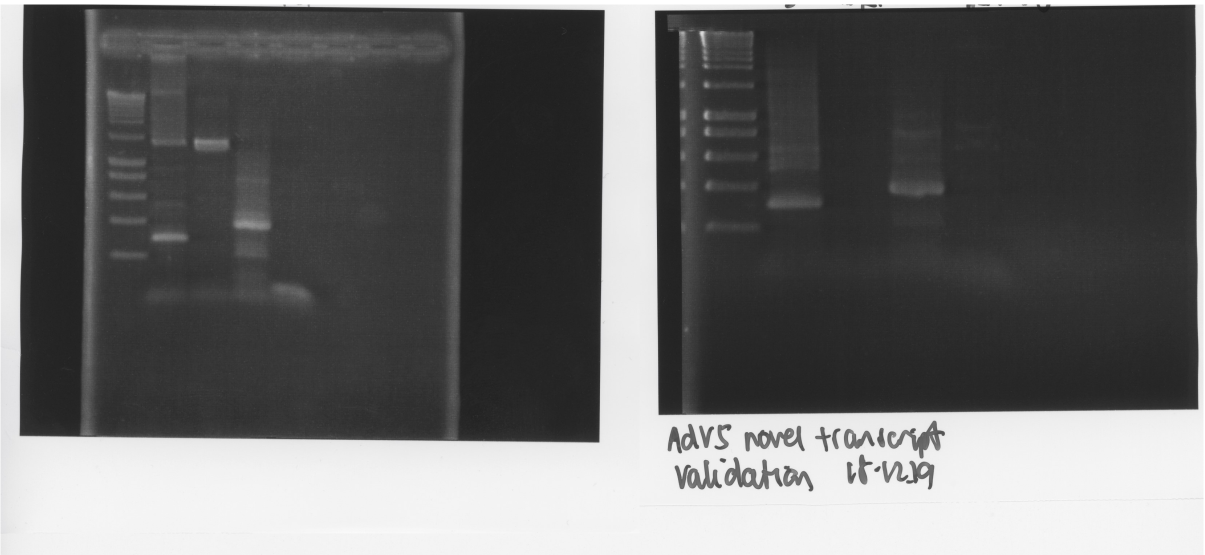

Supplementary Figure 1
Original gels used to demonstrate targeted RT-PCR. Gel on the left was used to validate targets 1 and 2, the gel on the right was used to validate targets 3 and 4.

Supplementary Tables

	16 hours post infection	24 hours post infection	48 hours post infection
Total number reads passed QC	2,219,024	1,545,410	1,834,888
Longest read	23,127nt	18,205nt	17,678nt
Average length	1,515nt	1,485nt	1,553nt
Total reads mapping to adenovirus genome	42,412 (1.9%)	186,712 (12.1%)	873,929 (47.6%)
Longest transcript mapped to adenovirus	13,848nt	13,160nt	16,089nt
Most abundant read length mapped to the genome using a 100nt window	1,800 - 1,900nt long (6,315 reads)	1,800 - 1,900nt long (16,444 reads)	1,900 - 2,000nt (75,720 reads).
Total reads mapping to adenovirus genome with poly A tail length measured >20 nt	25,000	114,072	555,236
Percentage of adenovirus-mapped transcripts coding for early proteins (E1-4)	50.3%	18%	4.3%
Percentage of adenovirus-mapped transcripts coding for late proteins (L1-5)	12.3%	38.7%	47.1%

Supplementary Table 1

Characterisation of nanopore sequencing outputs and mapping data at each timepoint post-infection with human adenovirus type 5.

The number of total reads that passed QC are listed for each time post-infection alongside the longest and average read lengths obtained, total reads mapping to the virus genome after error correction (as a percent of all reads), longest read length and the most abundant read length window that mapped to the viral genome (number of reads in that window in brackets). We also list how many reads had a measured polyadenylation length of at least 20nt and passed the QC checks during nanopolish polyA analysis. Finally, we provide basic information of the percentage of adenovirus mapped reads (corrected and with polyA > 20) that were identified as likely coding for an early or late protein because the transcript's 5' most ORF had been positively identified as a known adenovirus ORF.

Gene	Previously published TSS location	Dominant TSS location	Total reads mapped to the dominant TSS (number soft clipped)	Average 5' soft clip length (modal soft clip length; reads with that soft clip length)	Nearest 'AAUAAA' TTS signal (location refers to 'U' in the sequence)	Dominant TTS location	Total reads mapped to the dominant TTS (number soft clipped)	Average 3' soft clip (modal soft clip length; reads with that soft clip length)
E1a	499	511	417 (1)	1 (1; 1)	1613	1,630	259 (252)	24 (3; 47)
E1b	1,702	1,715	3,976 (150)	42 (1; 59)	4040	4,073	8,732 (8,719)	24 (5; 1378)
L1 (MLP)	6,049	6,061	130,725 (13,209)	5 (1; 8023)	14099	14,124	4,678 (4,670)	20 (6; 823)
L2 (MLP)					17,953	17,972	12,592 (12,592)	30 (8; 1712)
L3 (MLP)					22,374	22,386	12,867 (12,309)	36 (2; 2293)
L4 (MLP)					28,166	28,161	18,045 (17,677)	24 (2; 3574)
L4 (L4 promoter)	26,114	26,128	348 (22)	12 (1; 16)				
L5 (MLP)	6,049	6,061	130,725 (13,209)	5 (1; 8023)	32,785	32,790	16,022 (15,374)	26 (2; 2705)
E3A	27,568	27,582	4,813 (105)	22 (2; 34)	29,774 (AUUAAA)	29,788	1,429 (1,427)	18 (1; 303)
E3B					30,840	30,825	1,996 (1,996)	19 (1; 308)
E2 'early TSS' (DBP/pTP/Pol TSS)	27,051	27,036	5,085 (4732)	2 (3; 2223)	22,391	22,374	7,225 (7,222)	29 (9; 526)
E2 'late TSS' (DBP)	25,908	25,897	1,841 (552)	2 (1; 312)				
IVa2 (pTP/Pol TTS)	5,838	5,826	3,784 (83)	7 (1; 55)	4,093	4,057	1769 (1,753)	21 (4, 324)
E4	35,611	35,596	744 (445)	3 (5; 84)	32,819	32,825	485 (350)	24 (2; 87)
UXP (shares TTS with DBP)	31,062	31,050	497 (11)	2 (1; 5)	22,391	22,374	7,225 (7,222)	29 (9; 526)
pIX	3,582	3,590	14,722 (1,038)	5 (2; 466)	4,040	4,073	8,732 (8,719)	24 (5, 1378)

Supplementary Table 2

<u>Characterisation of nanopore TSS and TTS mapping locations.</u>

Transcript data from all three infected cell RNA timepoints was collated and the dominant locations of transcription start sites (TSS) and transcription termination sites (TTS) were located and analysed relative to well established TSS sites for adenovirus genes and the nearest AAUAAA transcription termination signal. In addition, we noted how frequently transcripts were soft clipped by the mapping software (minimap2) when the transcript was mapped to the viral genome. Soft clipping refers to instances where the mapping software cannot align regions at either (or both) the 5' or 3' end of a sequence to the target genome and so these regions are "soft clipped" so that they do not form part of the mapping information. For each site we noted the number of times transcripts were soft clipped: of those that were soft clipped we then calculated the average soft clip length and noted the most frequent soft clipping event (the modal) and how many transcripts were soft clipped by the modal amount.

ORF coded for	Count 16hpi	Percent of total 16hpi	Average poly A length 16hpi	Count 24hpi	Percent of total 24hpi	Average poly A length 24hpi	Count 48hpi	Percent of total 48hpi	Average poly A length 48hpi
52/55K(L1)	259	1.04	163	2668	2.34	123	6829	1.23	75
preIIIa(L1)	40	0.16	194	1476	1.30	136	4335	0.78	72
**TOTAL L1**		**1.20**			**3.64**			**2.01**	
preVII(L2)	357	1.43	144	5814	5.10	99	17351	3.13	80
pV(L2)	218	0.87	130	3945	3.46	98	12798	2.31	78
preX(L2)	183	0.73	158	5556	4.87	126	44857	8.09	83
Penton base(L2)	165	0.66	139	2386	2.09	109	7595	1.37	90
**TOTAL L2**		**3.69**			**15.52**			**14.90**	
hexon(L3)	276	1.10	186	4891	4.29	149	42736	7.71	101
preVI(L3)	45	0.18	184	1087	0.95	144	10420	1.88	106
23K-protease(L3)	31	0.12	199	472	0.41	129	5480	0.99	91
**TOTAL L3**		**1.40**			**5.66**			**10.58**	
100K(L4)	612	2.44	152	4147	3.64	117	13062	2.36	84
33K (L4)	482	1.92	142	4385	3.85	108	19658	3.55	73
33K 2nd exon(L4)	174	0.70	147	2116	1.86	121	13403	2.41	81
22K(L4)	98	0.39	87	347	0.30	83	1341	0.24	73
preVIII(L4)	67	0.27	142	767	0.67	127	6499	1.17	79
**TOTAL L4**		**5.73**			**10.3**			**9.73**	
fibre(L5)	242	**0.97**	174	6262	**5.49**	130	68141	**12.29**	89

Supplementary Table 3

MLTU gene expression and average poly A lengths.

Raw numbers of counts for RNAs encoding each ORF, and percent of total transcripts mapping to the viral genome at each timepoint these represent. Percentages do not sum to 100% as non-classical ORF-coding transcripts and transcripts that do not apparently code for anything are not included. In each case the average poly A length refers to the average length on the dominant transcript group coding for that protein. We have also summed the percentages for each late class of transcript; note that for L5 the fibre is the only ORF coded for by that class.

	i-leader exon plus TPL3 and then spliced to the indicated ORF	tpl 1,2,3 exons without i-leader then spliced to the indicated ORF	Percent inclusion of i-leader	PolyA length with i-leader, polyA length without i-leader
52/55K protein (L1)	2664	8557	31.13	103, 93
preIIIa (L1)	455	4943	9.20	108, 85
penton base (L2)	371	8929	4.16	101, 95
preVII (L2)	266	17410	1.53	103, 83
pV (L2)	209	12886	1.62	93, 80
preX (L2)	758	34004	2.23	99, 90
preVI (L3)	167	10360	1.61	125, 110
hexon (L3)	872	41649	2.09	121, 108
23K protease (L3)	127	3862	3.29	94,95
100K (L4)	784	15603	5.02	93,92
22K (L4)	11	558	1.97	74, 80
33K (L4)	152	23469	0.65	103,81
preVIII (L4)	20	2871	0.70	84,82
Fibre (L5)	301	38331	0.79	121, 93
y-leader Fibre (L5)	44	26600	0.17	142, 93

Supplementary Table 4

Inclusion of i-leader exon in major late transcripts.

Numbers of transcripts belonging to each transcript group with a TSS at the major late promoter, containing the tripartite leader exons and spliced to a major late ORF such that that the indicated ORF is the 5' most ORF, in comparison to numbers of equivalent transcripts that contain the i-leader exon between TPL2 and TPL3. Percent inclusion of i-leader is also shown and the average polyA lengths of transcript groups with and without i-leader exons.

Time p.i.	L4 22K transcripts				L4 33K transcripts			
	L4P-derived		MLP-derived		L4P-derived		MLP-derived	
	Number	%	Number	%	Number	%	Number	%
16 h	59	60.8	38	39.2	20	4.75	401	95.2
24 h	144	42.0	199	58.0	98	2.46	3885	97.5
48 h	198	15.2	1107	84.8	239	1.39	16988	98.6

Supplementary Table 5

Use of MLP or L4P to drive expression of 22K and 33K transcripts

Transcript counts were derived from Supplementary Tables 3, 5 and 7. Transcripts were assigned as 22K or 33K based on their first ORF, and all differences in transcript architecture distal to the ORF ignored to generate pooled transcript count values. L4P transcripts and MLP transcripts were those with 5’ ends at 26118 and 6051 respectively. The high level of MLP-derived transcription gave rise to groups of 5’ ends due to RNA breakage, distributed across the MLTU. Although at low level relative to intact MLP-derived RNAs, in the vicinity of the much weaker L4P, such 5’ ends might either be derived from MLP transcripts as above or else reflect alternative L4P initiation sites. The numbers of such transcripts were found to co-vary with the numbers of MLP-derived rather than L4P-derived L4 22K/33K transcripts and were therefore included in the MLP-derived transcript count.

Supplementary Notes, Discussion and Methods

Data analysis pipeline

The raw nanopore data was corrected using LorDEC (using the command line as stated in the methods) and a subset of the illumina sequence reads that were used in a paper from this group on integrative proteomics and transcriptomics. This short read data was from human cells infected with the same virus used in this paper and they were mapped to the viral genome and the mapping reads were turned back into fastq files before being normalised using the read normalisation tool in the Trinity package. This step is optional and is only used to reduce the size of the fastq files used for correction which will speed up the correction process. The LorDEC correction involved iterative rounds of correction as detailed in the methods section. The corrected reads were then mapped to the adenovirus genome with minimap (as in the methods section) and the resultant sam file (containing mapped reads only) was analysed by the in house scripts proved in the supplementary data.

The first step is to use "classify_transcripts_and_polya.pl" with the following command line:

classify_transcripts_and_polya.pl prefix polya/TSS_window splice_window polya_min nanopolish_polya.txt input.sam genome.fasta 1 100

prefix

Enter a short prefix that will help you remember what the outputs were for!

polya/TSS_window

Enter an integer for the size of window you want the software to use to bundle the transcription start or stop sites together (we used 15 in our analysis)

splice_window

Similarly for bundling splice sites together, normally not needed if the nanopore data has been corrected by illumina but can be useful if using uncorrected data and you have sufficient depth of read that a "true" splice site will rise above the noise.

polya_min

If you have polya.txt files from the nanopolish polya utility then you can specify here the minimum length of polya required alongside the "PASS" flag from nanopolish polya before a transcript will be analysed.

Nanopolish_polya.txt

If you have polya.txt files from the nanopolish polya utility then put the name of the files here, if you don't have this data the just put no_polya instead and the script will carry on without that data and just put a poly A length of -1 for everything.

Input.sam

Here you put the name of your sorted sam file.

Genome.fasta

Here you put the name of the file that contains the genome you mapped your data to

1

This is the minimum copy number of transcript group you want the software to consider, we used 1

100

This is the maximum number of entries you want to see in the gff file called "Prefix.gff_most_popular_list.gff3" file, this GFF3 file is useful in the early analysis steps to give you a broad overview of what is being made as it will return (in this example) only the top 100 most abundant transcript groups found.

The software will examine each transcript and note where the start and end locations of the mapped transcript along with the locations of splice acceptor and donor sites and the amount of soft clipping. This is used to group transcripts together if they have start and end locations within the window specified and if they have the same splice pattern. For example, once the start locations of all the transcripts are known the software then ranks each start location based on how many times it is used. The most used transcription start site is selected first and all other start sites for within your specified window are corrected so that they now all start at that location. Thus, all the transcript groups identified with a start location within that window will have their transcription start sites modified to reflect the dominant transcription start site. The outputs are:

prefix.canonical_transcripts_by_abundance.fasta
A list of pseudo transcripts generated by using the start, end and splice patterns of the transcript groups found, one transcript per transcript group along with information on how many individual reads belong to that transcript group, the average polyA length (if available) and the mapping co-ordinates.

prefix.GFF_all_found.gff3
A gff3 file describing each transcript group for visualisation in a viewer (e.g. IGV viewer).

prefix.GFF_most_popular_list.gff3
As above but only listing the 100 most abundant transcript groups.

prefix.raw_soft_clip_locations.txt
A tab separated list of the locations on the genome where there is soft clipping on either the plus or minus strand

prefix.start_sad_stop_pattern_count.txt
*A tab separated table listing all the transcript groups describing each groups start location, end location, strand, **s**plice **a**cceptor/**d**onor locations (referred to as the **sad** location) how many transcripts belong to that group and the average and standard deviation for the poly A length (where available). This file is used by the next software stepto analyse the genome and assign features and ORFS to the transcript groups.*

prefix.raw_processed_start_sad_polya.txt
*Tab separated list describing for each nucleotide position how often that is the location of a transcript start, a transcript end or a **s**plice **a**cceptor/**d**onor. Both the raw counted numbers are listed alongside the cumulative counts of events at each nucleotide location **after** the software has grouped nearby events together using the window size specified in the command line.*

Using **name_transcripts_and_track_ssc.pl** to find ORFs and determine what features are present on each transcript as defined by the transcript grouping software is the next step.

The command line is:

name_transcripts_and_track_ssc.pl prefix ssc_nt features_table.txt genome.fasta start_sad_stop_pattern_count.txt

prefix

Enter a short prefix that will help you remember what the outputs were for!

ssc_nt

Enter a value for ***s****tart* ***s****ite* ***c****orrection – the number of upstream nucleotides you want the pseudo transcript to include in order to account for the loss of 5' nucleotides in nanopore sequencing. We used a value of 10.*

features_table.txt

The name of the text file containing a list of features on the genome you are analysing (see below for format).

genome.fasta

The name of the fasta file with the viral genome.

start_sad_stop_pattern_count.txt

The name of the file from the first script that contains the information on the transcript groups

For this script to work, the user supplies a "features_table.txt" list of features, their locations on the viral genome and the name you want associated with each feature – conceptually similar to a GFF3 file but simpler. For example in each tab field in order you provide: the genome name<TAB>strand<TAB>location of the start codon<TAB>sad location<SPACE>sad location<… repeat as needed or leave blank if not needed…><TAB>location of stop codon<TAB> name of the feature.
Here is the entry for the ORF for the E1a 12S protein:

gi|56160529|ref|AC_000008.1| + 559 975 1229 1546 E1a_12S

This tells the software that between 559 and 975 there is an exon and between 1229 and 1546 there is an exon, the two should be connected and the transcript covered by these regions contains a feature called E1a_12S.
When adding a free text name to the feature please use underscore instead of space.
When describing features on the opposite strand, the entries for start and stop will be the actual locations of the start and stop, but the sad locations describing the exon/intron boundaries must be in numerical order:

gi|56160529|ref|AC_000008.1| - 14120 10590 14111 8583 preTP(E2B)

If you add the text "_TSS" then the software will understand that you are describing a transcription start site and any transcript group that has a start located within 50nt of that location will have this feature description added to the "features found" part of the output (see below).
This file is used by the software to generate pseudo transcripts based on the supplied coordinates. These pseudo transcripts are stored for comparison with your transcript groups later and they are also scanned 5' - 3' to determine if there is an AUG and if the sequence of the protein is 6aa or longer then it too is stored. If the ORF is stored then it is listed as a canonical ORF, a fasta file of the ORFS it thinks it has found in this way and the names

associated with them is produced. Next the software examines each transcript group in the same way, generating a pseudo transcript that is then examined to see if it contains any of the nucleotide sequences described by the features file. These features found and the splice acceptor/donor sequences at any intron/exon boundaries are also noted and reported. Allied to this the software then scans from 5' to 3' on the pseudo transcript looking for the first and second AUGs, both are translated and if the first AUG is the start codon for a canonical ORF then that is listed as the primary ORF. If the first AUG does not lead to a canonical ORF then the second ORF is examined to see if that one is the beginning of a canonical ORF. If neither are canonical ORFs then the primary ORF is reported alongside the transcript group and the transcript group is flagged as having no known ORFs and the transcript itself is added to the tab separated line of information about that transcript group.
The following files are produced:

prefix.TSS_start_sad_polyA_count_list.txt
This is a list (in descending order of number of observed transcripts belonging to each group) of all the transcript groups analysed.

prefix.proteins_known.fasta
A fasta file of the proteins the software found after analysing the canonical features list you provided.

prefix.proteins_not_known.fasta
A fasta file of proteins the software found after analysing the transcript groups that could not be matched to the list of canonical proteins.

prefix.most_abundant_trans_per_orf.gff
A GFF3 format file describing the most abundant transcript group that will code for each one of the canonical ORFs.

prefix.count_of_translated_features.txt
A tab delimited file detailing, for each ORF identified, how many transcripts in total would code for that ORF and (if available) the average poly A length for the dominant transcript group that codes for the indicated ORF.

prefix.combined_counts_and_feature_names.txt
A tab delimited file containing a suumary for each transcript group. The strand, start, sad locations and poly adenylation site alongside the number of observed transcripts belonging to that group. In addition, a unique name for each transcript group, the average polyA length and standard deviation, a list of features found on that transcript, the first ORF found, the second ORF found (if the first AUG does not code for a canonical ORF), the sequence of the first ORF and the sequence of the transcript if the transcript does not contain any recognised ORFs.

prefix (folder)
The software will also create a folder with the prefix used as the name for the folder. Inside the folder is a series of GFF3 files, one for each ORF found. Thus the user can examine the

GFF3 file for, say, the hexon protein and gain an overview of what transcript groups appear to code for that protein.

When the software tries to identify a canonical ORF on a transcript it will also look at some simple alternatives if the first AUG dies not code for a canonical ORF:
If the only canonical ORF found is initiated from the second methionine then the text "_2nd_M" will be added to the name of the ORF found.
If the ORF found contains a canonical ORF but the sequence has been extended/truncated then the text "possible 5'(or 3') truncation (or extension) of…" will be inserted into the description.

Summary of data processing steps

1. Obtain nanopore reads (we removed the reads mapping to human genes to reduce the size of the data and reduce compute times).
2. Obtain matched illumina data
3. Map illumina data to the adenovirus (or your organism's) genome and separate virus mapping reads from non-mapping reads then convert the mapping reads back to fastq data.
4. (OPTIONAL) use Trinity read normalisation to reduce the size of the illumina fastq files.
5. Use LorDEC to correct the nanopore data using the illumina data – we used repeated iterative rounds with increasing K-mer values. Any software that does this is fine to use.
6. Generate a features file as we have described for adenovirus for your organism (making sure the genome name match the SAM file is critical). The exact location of the starts and stops is not too important as the software will translate so you can use start and stop locations a few nucleotides either side if you want to.
7. Us the two perl scripts to find featires/ORF and characterise your data.

Comparing Nanopore splice sites to Illumina splice sites

For this analysis we used a SAM file of illumina reads that were mapped to the adenovirus genome and we compared it to the list of identified splicing events as listed in the **prefix.combined_counts_and_feature_names.txt** file produced by the pipeline above. The software included in the supplementary data is called `compare_nanopore_splices_to_illumina.pl` and it also requires a fasta file of the adenovirus genome. This software first analyses the illumina SAM file and identified splicing events and uses the viral genome to identify the splice acceptor/donor sites along with their location. This data is then compared to the list of splice sites present in the **prefix.combined_counts_and_feature_names.txt** file.

The command line is:
compare_nanopore_splices_to_illumina.pl prefix_2 mapped_illumina_reads.sam prefix.combined_counts_and_feature_names.txt genome.fasta

prefix
Enter a short prefix that will help you remember what the outputs were for!

mapped_illumina_reads.sam
This is the sam file format of your reads mapped to the target genome

prefix.combined_counts_and_feature_names.txt
Provide the counts and feature names text file generated in the earlier analysis of the nanopore data.

genome.fasta
A fasta file of the genome used to map the illumina data.

Three files are produced:

Prefix.illumina_juncs_and_summary.txt
This file lists the locations and splice acceptor donor pairs of all the splice events found in the illumina data and it also lists the *distinct* mapping sites for every illumina read that contains the names splice event, how many distinct start mapping sites were observed plus the total depth of reads that cover the named splice event.

Prefix.evidence_good.txt
This is a file that is in format the same as the **prefix.combined_counts_and_feature_names.txt** file but here are all the transcripts for which every splice event has evidence for it in the illumina file, the features column will have additional information added in the name of **FIIR** and **RIIIR** which stand for **F**orward **I**ndependent **I**llumina **R**eads and **R**everse **I**ndependent **I**llumina **R**eads. This is a count of how many reads cover the splice site that have unique mapped start sites and whether they are mapped to the forward or reverse strand.

Prefix.evidence_poor.txt

This is the same as above but containing all the transcripts that have at least one splice event that is not present in the illumina data. In addition, if the splice sites do not have canonical splice acceptor donor pairs (GU-AG, GC-AG, AT-AC or GT-GG) this will be flagged here.

Supplementary PCR methods

Exon-exon pairing number on Figure 10A	Sequence of primer	Short name	Number of copies of message observed[a]	Expected size of PCR product
	5’ CCATTGGAGGTAACTAGAGG 3’	Universal reverse primer for Fibre		N/A
1	5’ CAGAACAGGAGACTGCATTCC 3’	y-leader to novel exon fusion	43 + 10[b]	289nt
2	5’ TCTGGCGGAGTTTCAACCCAG 3’	long_tpl3 to y-leader fusion	41	378nt
3	5’ CAGCTGTTGGGTACTCTTGGATC 3’	tpl1 to short_tpl2	16[c] + 22	310nt[c]
4	5’ CCTCCGAACGTTTCAACCCAG 3’	tpl2 to y-leader fusion	357	378nt

List of RT-PCR primers used to validate novel exon-exon boundary findings from nanopore direct RNA sequencing. The universal reverse primer was used to prime the reverse transcription step (30 minutes at 50°C).

For each forward primer the nucleotides are coloured in red and black to indicate where the exon-exon boundary is.

The PCR used 50°C for the annealing temperature and 35 cycles.

[a] The number of copies of message described in Figure 10A, i.e. originating from the Major Late Promoter and with the fibre ORF as the 5’ most ORF.

[b] There are two transcripts in figure 10A that could explain this PCR product. Either would generate the same size PCR product.

[c] This assumes that the transcript without the y-leader is detected. Addition of the y leader would generate a 500bp product, there were 22 copies of this longer transcript.
