# Supplementary material for: Deep splicing plasticity of the human adenovirus type 5 transcriptome drives virus evolution
Source: Commun Biol. 2020 Mar 13;3:124. doi: 10.1038/s42003-020-0849-9 (PMC7070027; doi:10.1038/s42003-020-0849-9)
Supplement: Supplementary file 17 — Description of Additional Supplementary Files [file 42003_2020_849_MOESM17_ESM.pdf]

Description of additional supplementary items

Supplementary_Data_1_16hpi_TSS_analysis.xlsx
Analysis of transcription start sites at 16hpi

Supplementary_Data_2_24hpi_TSS_analysis.xlsx
Analysis of transcription start sites at 24hpi

Supplementary_Data_3_48hpi_TSS_analysis.xlsx
Analysis of transcription start sites at 48hpi

Supplementary_Data_4_Ad5_162448hpi.combined_counts_and_feature_names.xlsx
List of all identified transcript groups, each group is also tagged with known features from the virus transcriptome.

Supplementary_Data_5_16_24_48_time_course.xlsx
Table comparing the quantities of identified transcripts at each time point and polyA lengths. Also included here is the statistical analysis of the polyA lengths.

Supplementary_Data_6_A549_Ad5_compare_to_nanopore.illumina_juncs_and_summary.xlsx
List of locations and observation frequencies for illumina identified splice sites together with notation on whether they were also observed by nanopore and how many independent illumina reads confirm each site

Supplementary_Data_7_A549_Ad5_compare_to_nanopore.evidence_poor.xlsx
List of nanopore transcripts where at least one splice site cannot be confirmed by illumina data.

Supplementary_Data_8_A549_Ad5_compare_to_nanopore.evidence_good.xlsx
List of nanopore transcripts where all the splice sites are confirmed by illumina data including notation on how many independent illumina reads confirm the nanopore observed splice sites.

Supplementary_data_9_Ad5_features_table.txt
List of features on the adenovirus genome used to annotate the transcripts in the software name_transcripts_and_track_ssc.pl

Supplementary_data_10_Ad5_162448hpi_most_abundant_trans_per_orf.gff
GFF file of the most abundant transcript for each known ORF across all timepoints

Supplementary_data_11_Ad5_48hpi_most_abundant_trans_per_orf.gff
GFF file of the most abundant transcript for each known ORF at 48 hpi

Supplementary_data_12_Ad5_24hpi_most_abundant_trans_per_orf.gff
GFF file of the most abundant transcript for each known ORF at 24 hpi

Supplementary_data_13_Ad5_16hpi_most_abundant_trans_per_orf.gff
GFF file of the most abundant transcript for each known ORF at 16 hpi

Supplementary_data_14_Ad5_162448hpi.count_of_translated_features.xlsx
A spreadsheet of the observed counts for each known ORF combining all three timepoints
